# The Relationship between Musculoskeletal Symptoms and Work-related Risk Factors in Hotel Workers

**DOI:** 10.1186/2052-4374-25-20

**Published:** 2013-10-11

**Authors:** Jin Woo Lee, Ju Jong Lee, Hyeon Je Mun, Kyung-Jae Lee, Joo Ja Kim

**Affiliations:** 1 Department of Occupational and Environmental Medicine, Soonchunhyang University Hospital, Seoul, South Korea

## Abstract

**Objectives:**

To identify work-related musculoskeletal symptoms and any associated work-related risk factors, focusing on structural labor factors among hotel workers.

**Methods:**

A total of 1,016 hotel workers (620 men and 396 women) were analyzed. The questionnaire surveyed participants’ socio-demographics, health-related behaviors, job-related factors, and work-related musculoskeletal symptoms. Work-related musculoskeletal symptoms were assessed using the Nordic musculoskeletal questionnaire. All analyses were stratified by gender, and multiple logistic regression modeling was used to determine associations between work-related musculoskeletal symptoms and work-related risk factors.

**Results:**

The risk of developing work-related musculoskeletal symptoms was 1.9 times higher among male workers in the kitchen department than males in the room department (OR = 1.92, 95% CI = 1.03-3.79), and 2.5 times higher among male workers with lower sleep satisfaction than those with higher sleep satisfaction (OR = 2.52, 95% CI = 1.57-4.04). All of the aforementioned cases demonstrated a statistically significant association with work-related musculoskeletal symptoms. Moreover, the risk of developing work-related musculoskeletal symptoms was 3.3 times higher among female workers aged between 30 and 34 than those aged 24 or younger (OR = 3.32, 95% CI = 1.56-7.04); 0.3 times higher among females in the back office department than those in the room department (OR = 0.34, 95% CI = 0.12-0.91); 1.6 times higher among females on shift schedules than those who were not (OR = 1.60, 95% CI = 1.02-2.59); 1.8 times higher among females who performed more intensive work than those who performed less intensive work (OR = 1.88, 95% CI = 1.17-3.02), and; 2.1 times higher among females with lower sleep satisfaction than those with higher sleep satisfaction (OR = 2.17, 95% CI = 1.34-3.50). All of the aforementioned cases also displayed a statistically significant association with work-related musculoskeletal symptoms.

**Conclusion:**

This study focused on structural risk factors in the working environment, such as the gender-based division of labor, shift work and labor intensity, that demonstrated a statistically significant correlation with the work-related musculoskeletal symptoms of hotel workers. Both men and women reported different prevalence rates of work-related musculoskeletal symptoms among different departments. This could indicate that a gender-based division of labor produces different ergonomic risk factors for each gender group. However, only females displayed a statistically significant correlation between shift work and labor intensity and musculoskeletal symptoms. Thus, minimizing ergonomic risk factors alone does not suffice to effectively prevent musculoskeletal diseases among hotel workers. Instead, work assignments should be based on gender, department, working hours and work intensity should be adjusted to address multi-dimensional musculoskeletal risk factors. In addition, an approach that seeks to minimize shift work is needed to reduce the incidence of musculoskeletal disorders.

## Introduction

Work-related musculoskeletal disorders had been believed to be mostly associated with laborers in the manufacturing industry, who often work near conveyor belts at a fixed pace or in a repetitive manner. In recent years, however, a changing industrial structure across all sectors has increased the incidence of work-related musculoskeletal disorders, especially in the hospital, hotel, retail, clerical, financial, and educational sectors [1]. The first study on work-related musculoskeletal disorders in South Korea was conducted in 1989, and was entitled “Cervicobrachial disorders of female international telephone operators” [2]. The ensuing research focused on white-collar workers who performed work at visual display terminals (VDTs) work [3] and blue-collar workers in the construction [4], shipbuilding [5], and automobile industries [6]. As work-related musculoskeletal disorders garnered more understanding and attention, research subjects expanded to encompass diverse laborers such as hair stylists [7], medical workers at hospitals [8], golf caddies [9], and street cleaners [10].

The hotel business is one of the major services in the hospitality industry. Around 40% of employees at hotels are women, higher than the proportion in other sectors in Korea [11]. In addition, behaviors or attitudes of hotels’ employees play a decisive role in determining quality of service because the employees come in direct contact with customers when providing services. Consequently, the hotel business heavily relies on employees’ work capacity.

In the United States, the hotel and catering industry reported the second highest level of musculoskeletal risk factors behind the manufacturing sector [12]. Hotel workers in the U.S. were found to have been exposed to the following ergonomic risk factors that are known to be cause work-related musculoskeletal disorders: repeatability, excessive force, unnatural and static postures, and carrying heavy objects [13].

In addition to ergonomic risk factors, hotel employees work on a shift basis, as hotels operate 24/7. Moreover, hotel employees are exposed to the additional risk of relatively longer working hours [14,15], to a varying extent depending on the department and workplace. Hotels are businesses that provide services, and their employees serve customers face-to-face, requiring them to perform emotional labor with its increased mental burden, which is also known to be one of the precipitating factors of work-related musculoskeletal disorders [16]. Despite such findings, however, research on work-related musculoskeletal disorders among hotel workers has targeted only employees in certain divisions, such as chefs [17] and room maids [18], and none have addressed the entire range of hotel workers. In addition, given that hotel workers comprise a higher share of women than other worker groups, a separate study is required to explore gender-based differences and factors.

South Korea has launched the first move toward the management of work-related musculoskeletal disorders in 2003 with the enactment of Chapter 13 the “Occupational Health Standards,” entitled “Prevention of Musculoskeletal Burden-associated Health Damage.” A study that analyzed the effectiveness of Chapter 13 on the prevention of musculoskeletal disorders [19] in 2011 revealed that the manufacturing sector demonstrated improvement in such prevention, as 67.6% of manufacturers had examined hazards, while 59.4% had improved their working environment. In contrast, only 26.1% of companies in the retail and distribution, service, and education sectors had examined hazards and 22.7% improved their working environment. This indicates that Chapter 13 prevention system has yet to be widely adopted in industry, with the exception of the manufacturing sector. The hotel industry has long compelled workers to perform tasks that involve a musculoskeletal burden. However, their work is seen as imposing no such burden by the 11 standards stipulated under Chapter 13 because the workers take on a set of complex burdens while performing repetitive jobs that last for 2 to 4 hours, shorter than the legally defined period. Therefore, the current law’s standards are inadequate to gauge the level of the musculoskeletal burden affecting workers in the hotel business and develop measures to prevent the incidence of work-related musculoskeletal disorders.

Hotel workers are exposed to structural factors concerning working conditions associated with work-related musculoskeletal disorders, such as shift work, intensive labor, and a gender-based division of labor. This study aimed to identify the correlations between the incidence of musculoskeletal disorders among hotel workers and the traits of hotel workers’ labor.

## Methods

### Data

This study surveyed 1,320 workers of 5 hotels in Seoul from June 1, 2011 to October 21 of the same year. Among them, 1,072 workers participated in the survey (a response rate of 81.2%), and 56 respondents provided incomplete answers. As a result, this study analyzed the responses of 1,016 participants.

This study analyzed a total of 1,016 hotel workers (620 males and 396 females). The survey’s questionnaire asked respondents about their socio-demographics, health-related behaviors, job-related factors, and work-related musculoskeletal symptoms. Work-related musculoskeletal symptoms were assessed by using the Nordic musculoskeletal questionnaire. Based on a gender-stratified analysis, multiple logistic regression modeling was used to determine associations between musculoskeletal symptoms and work-related risk factors.

### Measures

#### Independent variables

Structured questionnaires were distributed to respondents for self-administration to discover the general and occupational traits of respondents and degrees of their work-related musculoskeletal symptoms. Respondents were classified into those who currently engage in drinking, smoking, or regular exercise, and those who did not. The research subjects were also distinguished in terms of daily sleeping hours, between those who reported sleeping 6 hours or longer, and those who slept less than 6 hours. The research subjects were additionally divided into those who reported getting enough sleep to recover from fatigue and those did not. The hotel workers were grouped in terms of marital status, distinguishing between those who were married, and those who were not married due to divorce or bereavement or who were separated.

Hotels consist of departments responsible for rooms, food and beverage, and general management. The room department performs services related to the front desk, door, and bell desk, as well as housekeeping. The food and beverage department is made up of kitchen and food and beverage divisions (the latter division is staffed by food servers). Based on the aforementioned departments, this study categorized work areas into rooms, housekeeping, kitchen, food and beverage, facility maintenance, back office and other departments. On the basis of average weekly working hours, hotel workers were grouped into those who worked less than 45 hours per week, and those who worked 45 hours or longer. In terms of the employment period, they were classified into three groups: those who had worked for less than 10 years, those who had worked at least 10 years less than 20 years, and those who had worked 20 years or longer. The hotel workers were classified into those who worked on a shift schedule and those who did not. The survey respondents were asked to give a “yes” or “no” answer to the question, “Do you work overtime (longer than the legally defined 8 hours a day)?” to classify workers into groups of those who worked overtime (more than 8 hours) and those who did not. Regarding the question, “How intensive do you think your work is?”, those who responded “easygoing” (response 1) or “appropriate” (response 2) were bracketed together into employees who performed lower intensity work, and those who said “somewhat burdensome” (response 3) or “very burdensome” (response 4) into employees of higher intensity work.

#### Evaluation of work-related musculoskeletal symptoms

We used the Nordic musculoskeletal questionnaire (NMQ), developed by Kuorinka in 1987, to assess the quality of musculoskeletal symptoms. We translated the NMQ into Korean, and added the visual analogue scale (VAS) to measure the intensity of the symptoms. The NMQ has advantages compared to other survey methods, as respondents find it easier to complete the NMQ’s simpler questionnaire, and the NMQ’s standardized results make comparison more straightforward when conducting epidemiological studies. We asked the study participants, “Have you suffered an ache, pain, discomfort, or numbness of your neck, shoulder, elbow, wrist/hand, back, lower back, hip/thigh, knee, or ankle/foot during the last 12 months?” In addition, we inquired “Have you experienced any trouble carrying out daily activities other than work (e.g. housework and hobbies) because of the physical difficulty during the last 12 months?”, and “Have you had any physical difficulty during the last 7 days?” The respondents were then requested to gauge their pain on a 10-point scale from 0 to 9 points. Among the respondents who experienced any symptom in any of the 9 body areas in the past 12 months, we determined those who suffering symptoms with a pain score of five or more points in the last week to be positive criteria for musculoskeletal symptoms.

### Data analysis

All of the analyses were stratified by gender. The χ [2]-test was carried out to examine differences in general and occupational characteristics between the male and female workers, and to determine the distribution of such traits among the group of respondents who reported musculoskeletal symptoms and the group of those who did not. We first identified statistically significant general and occupational variables via the χ [2]-test, or univariate analysis, and then set the variables as independent variables and musculoskeletal symptoms as dependent variables to perform multiple logistic regression analysis. We used SPSS software version 14.0 (SPSS, Inc., Chicago, IL, USA) to execute the analysis.

## Results

### Gender characteristics of the study subjects

The 1,016 study subjects consisted of 620 males (61.0%) and 396 females (39.0%), with an average age of 37.2 (the average age for the males stood at 39.3 and that of the females, at 33.9). The analysis of general characteristics found the proportion of female respondents was significantly higher in the younger age group (p < 0.001); the group with unmarried status than the group with married status (p < 0.001); the non-smokers’ group than the smokers’ group (p < 0.001); the non-drinkers’ group than the drinkers’ group (p < 0.001); the group of those who did not exercise regularly than the group of those who did exercise regularly (p < 0.001); and the group of those who did not sleep well than the group of those who did. On the other hand, the female respondents did not show a significant difference according to sleeping time (Table 1).

**Table 1:** General characteristics of the study subjects

**Characteristics**	**Total**		**Male**		**Female**		p^*^
	**N**	**(%)**	**N**	**(%)**	**N**	**(%)**	
Age (years)							
≦24	112	(11.0)	34	(5.5)	78	(19.7)	<0.001
25-29	216	(21.3)	108	(17.4)	108	(43.4)	
30-34	162	(15.9)	98	(15.8)	64	(18.2)	
35-39	148	(14.6)	99	(16.0)	49	(18.7)	
40-44	109	(10.7)	86	(13.9)	23	(18.7)	
≧45	269	(26.5)	195	(31.5)	74	(18.7)	
Marital status							
Married	524	(51.6)	373	(60.2)	151	(38.1)	<0.001
Unmarried	492	(48.4)	247	(39.8)	245	(61.9)	
Smoking							
Yes	336	(33.1)	319	(51.5)	17	(4.3)	<0.001
No	680	(66.9)	301	(48.5)	379	(95.7)	
Alcohol drinking							
Yes	787	(77.5)	528	(85.2)	259	(65.4)	<0.001
No	229	(22.5)	92	(14.8)	137	(34.6)	
Physical activity							
Yes	734	(72.2)	482	(77.7)	252	(63.6)	<0.001
No	282	(27.8)	138	(22.3)	144	(36.4)	
Daily hours of sleep							
≧6	655	(64.5)	406	(65.5)	249	(62.9)	0.397
<6	361	(35.5)	214	(34.5)	147	(37.1)	
Satisfaction with sleep							
Yes	533	(52.5)	363	(58.5)	170	(42.9)	<0.001
No	483	(47.5)	257	(41.5)	226	(57.1)	
Total	1016	(100.0)	620	(100.0)	396	(100.0)	

^*^ By chi-squared test.

The analysis of occupational characteristics revealed that the food and beverage department had the largest number of workers, 312 persons, or 30.7%, while 487 employees, the highest share, at 48.0%, reported an employment period of less than 10 years. The respondents who worked more than 45 hours per week accounted for the largest proportion, at 65.5%, or 665 workers. Those who worked on a shift schedule numbered 587 participants, comprising 57.8%. The number of respondents who worked overtime came to 514, making up 50.6%, while 421 or 41.4% reported higher work intensity. 127 respondents, or 12.5%, said they had experienced taking a leave of absence or early leave. The proportion of females was significantly higher in the room, food and beverage, housekeeping, and back-office departments (p < 0.001) than the kitchen and facility maintenance departments; the group of employees with a shorter employment period (p < 0.001); the group of employees who did not work overtime (p = 0.048); and the group of employees who had taken an absence or early leave (P < 0.001). On the other hand, neither gender showed a significant difference in terms of weekly working hours, shift work, and work intensity (Table 2).

**Table 2:** Work related characteristics of the study subjects

** Characteristics**		**Total**		**Male**		**Female**	P^*^
	**N**	**(%)**	**N**	**(%)**	**N**	**(%)**	
Department							
Rooms	196	(19.3)	97	(15.6)	99	(25.0)	<0.001
Food & Beverage	312	(30.7)	157	(25.4)	155	(39.2)	
Kitchen	236	(23.2)	201	(32.4)	35	(8.8)	
Housekeeping	53	(5.2)	25	(4.0)	28	(7.1)	
Engineering	80	(7.9)	78	(12.6)	2	(0.5)	
Back office	139	(13.7)	62	(10.0)	77	(19.4)	
Work duration (years)							
<10	487	(48.0)	243	(39.2)	244	(61.6)	<0.001
10-19	295	(29.0)	189	(30.5)	106	(26.8)	
≧20	234	(23.0)	188	(30.3)	46	(11.6)	
Weekly working hours							
<45	351	(34.5)	227	(36.6)	124	(31.3)	0.083
≧45	665	(65.5)	393	(63.4)	272	(68.7)	
Shift work							
Yes	587	(57.8)	352	(56.8)	235	(59.3)	0.419
No	429	(42.2)	268	(43.2)	161	(40.7)	
Overtime work							
Yes	514	(50.6)	329	(53.1)	185	(46.7)	0.048
No	502	(49.4)	291	(46.9)	211	(53.3)	
Work intensity							
High	421	(41.4)	244	(39.4)	177	(44.7)	0.092
Low	595	(58.6)	376	(60.6)	219	(55.3)	
Absence / early leaves							
Yes	127	(12.5)	46	(7.4)	81	(20.5)	<0.001
No	889	(87.5)	574	(92.6)	315	(79.5)	
Total	1016	(100.0)	620	(100.0)	396	(100.0)	

^*^ By chi-squared test.

### Comparison of musculoskeletal symptoms in terms of gender and general and occupational characteristics

Among all of the respondents, 235 workers, or 23.1%, reported musculoskeletal symptoms. Of the workers with symptoms, 16.8%, or 104 people, were males, while 33.1% of them, or 131, were females. An examination of the relationship between general and occupational characteristics and musculoskeletal symptoms uncovered that the following variables were statistically significant among both males and females: age, subjective sleep satisfaction, department, overtime, work intensity, and absence or early leave. However, unlike the males, the female workers were found to have two additional statistically significant variables of weekly working hours and shift work (Table 3).

**Table 3:** Comparison of work-related musculoskeletal symptoms(WRMSs) by general and work related characteristics of the study subjects

** Characteristics**	**Male (n = 620)**				**Female (n = 396)**			
		**WRMSs(+)**		**WRMSs(-)**		**WRMSs(+)**		**WRMSs(-)**
	**N**	**(%)**	**N**	**(%)**	**N**	**(%)**	**N**	**(%)**
Age (years)								
≦24	2	( 5.9)	32	(94.1)^*^	21	(26.9)	57	(73.1)^*^
25-29	18	(16.7)	90	(83.3)	47	(43.5)	61	(56.5)
30-34	24	(24.5)	74	(75.5)	23	(35.9)	41	(64.1)
35-39	27	(27.3)	72	(72.7)	19	(38.8)	30	(61.2)
40-44	27	( 9.3)	78	(90.7)	8	(34.8)	15	(65.2)
≧45	8	(12.8)	170	(87.2)	13	(17.6)	61	(82.4)
Smoking								
Yes	47	(14.7)	272	(85.3)	8	(47.1)	9	(52.9)
No	57	(18.9)	244	(81.1)	123	(32.5)	256	(67.5)
Alcohol drinking								
Yes	86	(16.3)	442	(83.7)	93	(35.9)	166	(64.1)
No	18	(19.6)	74	(80.4)	38	(27.7)	99	(72.3)
Physical activity								
Yes	78	(16.2)	404	(83.8)	86	(34.1)	166	(65.9)
No	26	(18.8)	112	(81.2)	45	(31.3)	99	(68.8)
Daily hours of sleep								
≧6	64	(15.8)	342	(84.2)	83	(33.3)	166	(66.7)
<6	40	(18.7)	174	(81.3)	48	(32.7)	99	(67.3)
Satisfaction with sleep								
Yes	38	(10.5)	325	(89.5)^*^	37	(21.8)	133	(78.2)^*^
No	66	(25.7)	191	(74.3)	94	(41.6)	132	(58.4)
Department								
Rooms	14	(14.4)	83	(85.6)^*^	37	(37.4)	62	(62.5)^*^
Food & Beverage	25	(15.9)	132	(84.1)	63	(40.6)	92	(59.4)
Kitchen	48	(23.9)	153	(76.1)	7	(20.0)	28	(80.0)
Housekeeping	1	( 4.0)	24	(96.0)	7	(25.0)	21	(75.0)
Engineering	10	(12.8)	68	(87.2)	0	( 0.0)	2	(100.0)
Back office	6	( 9.7)	56	(90.3)	17	(22.1)	60	(77.9)
Work duration (years)								
<10	38	(15.6)	205	(84.4)	84	(34.4)	160	(65.6)
10-19	40	(21.2)	149	(78.8)	35	(33.0)	71	(67.0)
≧20	26	(13.8)	162	(86.2)	12	(26.1)	34	(73.9)
Weekly working hours								
<45	32	(14.1)	195	(85.9)	31	(25.0)	93	(75.0)^*^
≧45	72	(18.3)	321	(81.7)	100	(36.8)	172	(63.2)
Shift work								
Yes	64	(18.2)	288	(81.8)	87	(37.0)	148	(63.0)^*^
No	40	(14.9)	228	(85.1)	44	(27.3)	117	(72.7)
Overtime work								
Yes	67	(20.4)	262	(79.6)^*^	71	(38.4)	114	(61.6)^*^
No	37	(12.7)	254	(87.3)	60	(28.4)	151	(71.6)
Work intensity								
High	57	(23.4)	187	(76.6)^*^	73	(41.2)	104	(58.8)^*^
Low	47	(12.5)	329	(87.5)	58	(26.5)	161	(73.5)
Absence/early leaves								
Yes	16	(34.8)	30	(65.2)^*^	41	(50.6)	40	(49.4)^*^
No	88	(15.3)	486	(84.7)	90	(28.6)	225	(71.4)
Total	104	(100.0)	516	(100.0)	131	(100.0)	265	(100.0)

^*^ p < 0.05 by chi-squared test.

### Multiple logistic regression analysis of musculoskeletal symptoms by gender group

Following gender stratification analysis, we applied multiple logistic regression modeling to determine correlations between musculoskeletal symptoms and work-related risk factors. We adopted the following significant variables of the previous univariate analysis as control variables: age, subjective sleep satisfaction, department, weekly working hours, shift work, overtime, and work intensity. We found that the risk of developing work-related musculoskeletal symptoms was 1.9 times higher among male workers in the kitchen department than males in the room department (OR = 1.92, 95% CI = 1.03-3.79), and 2.5 times higher among male workers with lower sleep satisfaction than those with higher sleep satisfaction (OR = 2.52, 95% CI = 1.57-4.04). All of the aforementioned cases demonstrated a statistically significant association with work-related musculoskeletal symptoms. Moreover, the risk of developing work-related musculoskeletal symptoms was 3.3 times higher among female workers aged between 30 and 34 than those aged 24 or younger (OR = 3.32, 95% CI = 1.56-7.04); 0.3 times higher among females in the back office department than those in the room department (OR = 0.34, 95% CI = 0.12-0.91); 1.6 times higher among females on shift schedules than those who were not (OR = 1.60, 95% CI = 1.02-2.59); 1.8 times higher among females who performed more intensive work than those who performed less intensive work (OR = 1.88, 95% CI = 1.17-3.02), and; 2.1 times higher among females with lower sleep satisfaction than those with higher sleep satisfaction (OR = 2.17, 95% CI = 1.34-3.50). All of the aforementioned cases also displayed a statistically significant association with work-related musculoskeletal symptoms (Table 4).

**Table 4:** Adjusted odds ratios and 95% confidence intervals for work-related musculoskeletal symptoms(WRMSs)* according to related factors

**Independent variables**		**Male WRMSs(+)**		**Female WRMSs(+)**	
		**OR**^**†**^	**95%CI**^**‡**^	**OR**^**†**^	**95%CI**^**‡**^
Age (years)	≦24	1.00		1.00	
	25-29	0.49	0.10-2.07	1.42	0.61-3.30
	30-34	1.26	0.64-2.47	3.32	1.56-7.04
	35-39	1.78	0.93-3.38	2.04	0.89-4.65
	40-44	2.12	1.13-3.98	2.10	0.87-5.08
	≧45	0.51	0.21-1.20	1.51	0.49-4.60
Department	Rooms	1.00		1.00	
	Food & Beverage	1.16	0.56-2.42	0.92	0.51-1.64
	Kitchen	1.92	1.03-3.79	0.34	0.12-0.91
	Housekeeping	0.29	0.03-2.44	0.35	0.17-0.73
	Engineering	1.01	0.40-2.54	4.43	0.37-4.20
	Back office	0.68	0.23-1.93	0.34	0.12-0.91
Shift work	No	1.00		1.00	
	Yes	0.94	0.58-1.54	1.60	1.02-2.59
Work intensity	Low	1.00		1.00	
	High	1.53	0.95-2.47	1.88	1.17-3.02
Satisfaction with sleep	Yes	1.00		1.00	
	No	2.52	1.57-4.04	2.17	1.34-3.50

Using multiple logistic regression analysis.

* Adjusted for age, department, shift work, work intensity, satisfaction with sleep, weekly working hours, overtime work.

^†^ Odds ratio, ^‡^ Confidence interval.

## Discussion

This study examined the correlations between hotel workers’ musculoskeletal symptoms and their occupational characteristics. Risk factors that contribute to the incidence of work-related musculoskeletal symptoms can be categorized into ergonomic risk factors, structural factors regarding the working environment, and individual factors (including gender and age). This study aimed to identify the relationship between work-related musculoskeletal disorders and risk factors, with a focus on structural working environment factors, and explored prevention methods.

The incidence of work-related musculoskeletal symptoms showed a significant difference between genders, as 104 males, 16.8% of all study subjects, reported symptoms, whereas 131 females (33.1%) did so. According to a study that surveyed the health status of Korean workers [20], a smaller number of female workers than their male counterparts have had their symptoms legally recognized as occupational injuries, yet female workers reported a 2.5 times higher incidence of work-related musculoskeletal disorders than males did. Another study on the incidence of musculoskeletal disorders and the public health management system [21] found that a significantly higher ratio of females reported work-related musculoskeletal symptoms than males did. Female workers have been known to be vulnerable to musculoskeletal disorders, but different studies cite different causes. One study, which examined 56 previous studies on work-related musculoskeletal disorders, noted that being a 'female’ was a risk factor for upper extremity musculoskeletal disorders, which stemmed from other work-related exposures, psychosocial factors, cultural factors, and biological differences [22]. Some studies have shown that female workers are more exposed to factors such as layoffs due to corporate restructuring, early retirement, temporary employment and job insecurity than male counterparts [23], and thus suffer from greater job stress, whereas other research concluded the burden of juggling between work and family exacerbates musculoskeletal disorders [24]. This study revealed, that in comparison to males, female workers endure more frequent shift work, longer weekly working hours, more intensive labor, and a shorter employment period (Table 2). Females suffer poorer working conditions than males do, which may have served as a risk factor for work-related musculoskeletal disorders. In the multivariate analysis, shift work and labor intensity had a statistically significant impact on the prevalence rate of work-related musculoskeletal symptoms only among female workers.

Among hotel workers in room and food and beverage departments, females reported a far higher prevalence of work-related musculoskeletal disorders. The gender disparity may have occurred because males and females in the same department perform different tasks under different job titles, creating discrepancies between risk factors for male and female workers. Female workers in the room department often arrange room reservations and guide customers and thus are exposed to emotional labor and long hours of standing up while working. On the other hand, males frequently serve as valet parkers, doormen, bellmen, porters who carry luggage, and limo-drivers. Hotels food and beverage departments, which primarily provide food and beverage services, employ managers and other personnel in management positions, as well as staff in sub-management positions such as room food servers, bartenders, and banquet servers. The sub-management employees stand up for long periods of time while working, serve food and beverages in unstable postures, and keep hotel halls clean. The waiters in the food and beverage department showed a relatively more equal division of labor between genders. However, the men primarily conducted work that carried lower ergonomic risk factors, including promoting banquet services, booking banquet reservations and management of banquet services. On the other hand, men working in the kitchen recorded a higher prevalence of work-related musculoskeletal disorders than women did. A multiple logistic regression analysis of males’ work-related musculoskeletal symptoms found that men in the working in kitchen were more than 1.9 times more likely to carry risks of musculoskeletal disorders than men working in the room department. Likewise, both males and females showed a difference in the prevalence rate of work-related musculoskeletal symptoms among departments. As a series of prior studies [25,26] have discovered, males and females working for the same department seemed to have experienced different exposures to ergonomic risk factors because men and women are expected to perform different gender roles, causing a division of labor based on gender.

The hotel industry relies on shift work more frequently than other industries, because it needs to respond to customer inquiries 24 hours a day. Of the research subjects, 57.8% worked on a shift schedule, which was above the average of 29.9% among their European counterparts surveyed in 2007 [27]. Survey research organizations in Korea and their sampling and weighting techniques vary significantly, resulting in a pool of partial, unreliable data on shift work. In three separate surveys [28], 50.0% of domestic hotels and restaurants said their operations were based on shift work in 2003, 0.3% in 2007, and 34.0% in 2011, showing extreme variations depending on the survey year. A similarly wide gap was observed in other polls on their working conditions [29], as 18.3% of respondents reported working on a shift schedule in 2006, but only 8.4% in 2010. This study’s subjects showed a higher ratio of workers on shift schedules compared with those of workers researched by other studies.

In this study, multiple logistic regression analysis revealed that women on shift schedules experienced statistically significantly higher numbers of work-related musculoskeletal symptoms. Research that found a relationship between shift work and musculoskeletal symptoms [30,31] outnumbered research that did not find such a relationship [32]. Shift work also increased work-related musculoskeletal disorders in a statistically significant manner, according to a retrospective cohort study [33]. One study observed that public health and medical workers’ musculoskeletal disorders increased in proportion to shift work, because shift work reduced relaxation hours and increased working hours [34]. In addition, a study of 1,163 nurses discovered that work-related musculoskeletal disorders were twice as common in a group of nurses who worked on a shift for two weeks or longer a month than a group of those who worked on a shift for shorter periods [35]. The hotel industry finds it impossible to completely eliminate shift work. Nonetheless hotels need to seek ways to minimize shift work to reduce the occurrence of work-related musculoskeletal disorders, as this study has demonstrated that the accommodation businesses have reported a higher ratio of workers on shift schedules.

The hotels’ higher reliance on shift work, at the same time, is also likely to cause sleep deprivation [36]. A lack of sleep has proven to induce physiological dysfunction and build up fatigue, a major cause of sleepiness during working and subsequent accidents, as well as increasing risks of employees’ work efficiency loss and accidents [37]. 47.5% of research subjects, or 483 people, felt dissatisfied with their sleep, and this group reported a statistically significantly higher number of work-related musculoskeletal symptoms than the group of workers who felt satisfied with their sleep. Multiple logistic regression analysis yielded a similar result, as the risk of work-related musculoskeletal symptoms more than doubled in both males and females who were dissatisfied with their sleep. Another study also concluded that sleep disorders weaken the capacity for physical recovery and diminish pain tolerance, inducing subjective pain [38]. However, this study had a cross-sectional design, and thus was capable of establishing an association between sleep satisfaction and work-related musculoskeletal symptoms, but incapable of verifying a causal relationship. One study set pain as an independent variable and sleep disorders as a dependent variable and demonstrated a statistically significant association, indicating a reverse causal relationship in which musculoskeletal pain causing sleep dissatisfaction could exist [39]. Further research is required to establish a causal relationship between sleep satisfaction and work-related musculoskeletal symptoms.

In this study, 421 workers, or 41.4%, belonged to the group of high work intensity, and both males and females in this group experienced a significantly higher incidence of work-related musculoskeletal symptoms. However, multiple logistic regression analysis, which weighs all variables, revealed that only females of the high work intensity group displayed a statistically significant incidence of work-related musculoskeletal symptoms (Table 4). Another study of hotel workers reached a similar conclusion, that the group of workers whose duties increased per hour reported a higher odds ratio of prevalence of work-related musculoskeletal symptoms than the group of workers with no hourly increase in duties [40]. Korean workers have faced a greater risk of musculoskeletal disorders since 1997, when Korea sought a bailout from the International Monetary Fund (IMF) during the height of the Asian financial crisis and the ensuing recession. The economic turmoil put workers under the pressure of more intensive labor, reduced break time due to overtime or extra work, and more frequent shift work and increased occupational stress. The hotel industry started cutting costs through layoffs and outsourcing. Following the financial crisis, 27 five-star hotels in Seoul reduced their combined workforce from 16,400 to 14,800 employees [41].

The leading musculoskeletal risk factors in the hotel business are repetitive motion, excessive force, and unnatural and static postures. Hotel workers are exposed to repetitive motion while preparing or cooking food, dishwashing, and changing beds. Hotel workers are exposed to excessive force when lifting heavy luggage, transporting and reloading ingredients, serving customers, and cleaning. Hotel workers are exposed to unnatural postures as they become constrained by the height of the front desk while receiving customers or performing VDT tasks, and by confined space while cleaning and rearranging bathrooms. Hotel employees’ are exposed to the static postures of standing up without any moving for a prolonged time at the front desk when standing by [42]. Hotel workers are exposed to a variety of ergonomic risk factors simultaneously, and in a survey performed outside Korea [43], more than 60% of workers at hotels and restaurants reported three or more symptoms of pain, and only 16% of the respondents felt no symptoms. As demonstrated above, hotel workers are already exposed to many musculoskeletal risk factors, and therefore the shortage of workers increases the labor intensity of the current workforce, further aggravating their musculoskeletal disorders. One study examined the relationship between labor intensity and work-related musculoskeletal disorders among workers who perform standardized tasks in the shipbuilding sector [5] and another study in the automobile sector [44]. A study in Korea targeted workers responsible for public facility management, who often conduct non-standardized tasks like hotel workers do. The research found that workers in the public facility management sector underwent restructuring following the financial crisis and structural changes in their working environment, such as lower numbers of co-workers and an increased workload from 1998 to 2000, and after such changes, the workers showed statistically significantly greater odds of having work-related musculoskeletal symptoms [45]. A prospective study on Finnish workers in the public sector also showed that restructuring compelled workers to perform more intensive physical labor, thus intensifying their work-related musculoskeletal symptoms [46]. The Korean hotel industry has seen a steady expansion of restructuring and outsourcing practices since the Asian financial crisis, and in the process hotel employees have endured an increased workload and pressure to perform extra labor. Therefore, structural factors need to be improved in a way that augments the working environment to ensure that working conditions and labor intensity do not deteriorate, in addition to the need to address ergonomic risk factors [47].

The first limitation of this study is that we did not examine workers dispatched by outsourcing companies to the hotel industry even though they are likely to be exposed to the risk factors of musculoskeletal disorders. According to an assessment of five-star hotels in Seoul in 2005 [48], employees dispatched by outsourcing companies accounted for as much high as 36.3% of the workforce. 26% of room maids at the five-star hotels were dispatched workers, and the proportion of dispatched workers also stood at more than 20% at the cleaning and equipment management divisions. This study analyzed 1,016 workers, and only 53 workers, or 5.2%, were in charge of housekeeping. Many five-star hotels hire full-time housekeepers to clean floors reserved for VIP customers, but other floors for general customers are cleaned by dispatched workers [49]. Likewise, this study analyzed only full-time housekeepers, and did not include dispatched housekeepers. Housekeeping, cleaning and equipment management are dismissed as simple manual labor, and thus employees in these three divisions, who are hired by outsourcing companies, are likely to be exposed to risk factors for musculoskeletal disorders. This means the study’s result may not represent work-related musculoskeletal disorders of all workers. The second limitation of this study is that the research did not target all hotels in Seoul, and therefore it is difficult to generalize this study’s conclusions to a national level. The third limitation of this study is that a self-administered survey was performed to analyze work-related musculoskeletal symptoms and absence or early leaves, relying on respondents’ recollection of their experience in the previous year, and thus leaving a possibility of memory bias and overstatements. The final limitation of this study was that the research was a cross-sectional study and thus inadequate for identifying a causal association between independent variables and dependent variables. The analysis of a causal relationship between the abovementioned factors requires a prospective study.

Despite these limitations, this study is a valuable addition to the scarce research on musculoskeletal disorders of hotel workers. This study conducted gender stratification analysis to identify the prevalence rates of work-related musculoskeletal symptoms and general and occupational traits of hotel workers across all departments. This study also analyzed relevant factors to provide essential data needed for developing precautionary measures against work-related musculoskeletal symptoms.

This study focused on structural risk factors regarding the working environment, such as the gender-based division of labor, shift work and labor intensity, which demonstrated a statistically significant correlation with the work-related musculoskeletal symptoms of hotel workers. Both gender groups of men and women reported different prevalence rates of work-related musculoskeletal symptoms among different departments. This could indicate gender-based division of labor produces different ergonomic risk factors for different gender groups. However, only females displayed a statistically significant correlation between shift work and labor intensity and musculoskeletal symptoms. Minimizing ergonomic risk factors alone does not suffice to effectively prevent musculoskeletal diseases among hotel workers. Instead, work assignments should be based on gender and department, and working hours and work intensity should be adjusted to address multi-dimensional musculoskeletal risk factors. In addition, an approach that seeks to minimize shift work is needed to reduce the incidence of musculoskeletal disorders.

## Competing interests

The authors declare that they have no competing interests.

## Authors’ contributions

All authors read and approved the final manuscript. This work was supported by the Soonchunhyang University Research Fund.
